# Relationship between attitudes toward COVID-19 infection, depression and anxiety: a cross-sectional survey in Japan

**DOI:** 10.1186/s12888-022-04474-1

**Published:** 2022-12-19

**Authors:** Megumi Hazumi, Emi Okazaki, Kentaro Usuda, Mayumi Kataoka, Daisuke Nishi

**Affiliations:** 1 Department of Public Mental Health, National Institute of Mental Health, National Center of Neurology and Psychiatry, Kodaira, Tokyo Japan; 2 Department of Sleep-Wake Disorder, National Center of Neurology and Psychiatry, Kodaira, Tokyo Japan; 3 Department of Mental Health, Graduate School of Medicine, The University of Tokyo, Bunkyo, Tokyo Japan

**Keywords:** COVID-19, Sequelae of COVID-19, Depression, Anxiety, Mental health, Psychological difficulties

## Abstract

**Background:**

Although negative attitudes are known to develop with experiences of COVID-19 infection, it remains unclear whether such attitudes contribute to depression and anxiety as sequelae of COVID-19. We aimed to investigate the relationships between attitude towards COVID-19 infection and post-COVID-19 depression and anxiety.

**Methods:**

A cross-sectional survey of COVID-19 recovered patients was conducted from July to September 2021 in Japan. Outcome variables, depression and anxiety were assessed using the Patient Health Questionnaire-9 and Generalized Anxiety Disorder-7); scores of 10 and above were identified as having symptoms of depression and anxiety, respectively. Exposure variables were whether participants were experiencing the following attitude strongly: threat to life due to COVID-19 infection, helplessness regarding COVID-19 infection, blaming a third party who did not restrain from going outside, blaming themselves for their COVID-19 infection, worry about spreading the infection to others, and self-stigma (Self-Stigma Scale-Short). Modified Poisson regression analyses were performed to analyze the findings.

**Results:**

A total of 6016 responses were included in the analyses. The proportion of depression was 19.88%, and anxiety was 11.47%. The threat of life due to COVID-19 infection, helplessness regarding COVID-19 infection, blaming oneself for their COVID-19 infection, and self-stigma were significantly associated with depression and anxiety after adjusting covariates. Blaming the third party who did not restrain from going outside was associated with anxiety. There was no association between the worry about spreading infection to others and depression or anxiety.

**Conclusion:**

Negative attitudes, including self-stigma with the experience of COVID-19 infection, were related to depression and anxiety. Further studies confirming whether countermeasures for preventing or decreasing the negative attitude towards COVID-19 infection mitigate these symptoms are needed.

## Background

COVID-19 infection is problematic in sequelae of COVID-19 after recovery. Psychiatric symptoms, such as depression and anxiety, are commonly observed as sequelae [1]. According to a meta-analysis, 19.2%—21.5% of COVID-19 recovered patients were diagnosed with depression, and 14%—44% were diagnosed with generalized anxiety disorder [2]. Moreover, four months post infection, 20.6% had depressive symptoms and 31.4% had anxiety symptoms [3] (Morin et al., 2021). These psychiatric symptoms are usually observed six to twelve months after the infection, and the proportion of individuals experiencing such symptoms is higher than those who were not infected with COVID-19. Depressive and anxiety symptoms appear to be long-term residuals [4–6]. Individuals with other infectious diseases do not have such high rates of residual depressive and anxiety symptoms [5, 7, 8]. Depressive and anxiety symptoms cause low quality of life and socio-economic loss [9–11]. Therefore, exploring the factors relating to depressive and anxiety symptoms in COVID-19 recovered patients is necessary for preventing or moderating these symptoms.

Both psychological and biological or demographic factors such as age, comorbidity, hospitalization, and physical complaints are likely to affect depressive and anxiety symptoms as sequelae of COVID-19 infection [12–21]. In fact, some psychological factors known for affecting depression and anxiety in the general population were also observed to be associated with such psychiatric symptoms in COVID-19 recovered patients. For example, a cross-sectional survey explains that psychological stress perceived after COVID-19 infection contributes to mental health problems in COVID-19 recovered patients [22, 23].

In addition to general psychological factors mentioned above, COVID-19-specific psychological factors possibly contribute to depressive and anxiety symptoms in COVID-19 recovered patients, although this relationship remains unclear. Specifically, qualitative studies have found that COVID-19-recovered patients often suffer from several attitudes, newly developed due to COVID-19 infection experiences such as COVID-19-related-stigma [24, 25], guilt of spreading the infection [24–26], threat of ambiguity in prognosis [27], and feeling of distancing from others [27]. However, because quantitative research has been insufficient except for self-stigma [28–30], the degree of relationships between such attitudes toward COVID-19 infection and psychiatric symptoms such as depression and anxiety remains unclear. Therefore, we aimed to exploratorily investigate whether the attitudes toward COVID-19 infection were associated with depression and anxiety in COVID-19-recovered patients with the cross-sectional design.

## Methods

### Design and procedure

This study for COVID-19 recovered was conducted with the cross-sectional design using the online survey system.

The data collection of this study was conducted from July to September 2021 in Japan. All methods were performed in accordance with the relevant guidelines and regulations. [31]. Participants were recruited via an internet survey agency, Rakuten Insight, which is one of the major survey agencies in Japan with a panel of approximately 2.2 million in 2019 (Rakuten Insight, Tokyo, Japan). Participants who agreed to participate in the survey and met the inclusion criteria were asked to complete the questionnaire. Participants who responded “yes” to the screening question “Have you ever been infected with COVID-19?” were defined as meeting the inclusion criteria in this study. Among these participants, the following were excluded from the analyses: (a) participants who answered the dummy question incorrectly; (b) participants who disclosed not meeting the inclusion criteria; (c) participants whose duration after COVID-19 infection was outside the range of 0 to 20 months, based on the date of COVID-19 pandemic in Japan; (d) participants who selected “others” for the question on educational level and could not be reclassified based on open-ended responses. Except for the variable about duration after COVID-19 infection, no missing values and outlier values were found since the online survey system prevents proceeding if there are any missing or outlier responses.

### Patient and public involvement

One person who experienced COVID-19 infection participated in this study as a researcher. The psychological conflicts he experienced due to the COVID-19 infection were listed comprehensively, based on discussion between all researchers, and used as independent variables in this study.

### Measurement

#### Outcome variables

The Patient Health Questionnaire-9 (PHQ-9) was used to measure the presence of depression as it has high validity and reliability [32, 33]. It comprises nine items, rated on a 4-point scale: (0) not at all, (1) several days, (2) more than half the days, and (3) nearly every day. The Generalized Anxiety Disorder-7 (GAD-7) [34, 35] was used to measure anxiety. GAD-7 has also confirmed validity and reliability [34, 35], and comprises seven items rated on the same 4-point scale as the PHQ-9. A higher total score indicates greater symptom severity in both scales, and participants whose score were 10 and above were defined as having symptoms. [32–35].

#### Exposure variables

Based on the researcher’s experiences who experienced COVID-19 infection as mentioned above, the following questions were generated as attitudes specific to COVID-19 infection: “How strongly did you feel the threat of life due to COVID-19 infection?” “How strongly did you feel helplessness to COVID-19 infection?” “How strongly did you blame the third party, such as policy and those who did not restrain from going outside, for your COVID-19 infection?” “How strongly did you blame yourself for your COVID-19 infection?” “How strongly did you worry about spreading COVID-19 to others when you were infected?” These questions were evaluated on five levels: not at all, a little, fair, strong, and very strong. Participants who selected “strong” or “very strong” were classified as those who experienced these cognitions and emotions strongly, while those who chose the other three alternatives were classified as those who did not experience them strongly.

Self-Stigma Scale-Short (SSS-S) [36] was used to measure the severity of self-stigma related to COVID-19. This scale is short version of the Self-Stigma Scale (SSS) with sufficient validity and reliability [36, 37]. The scale is designed to measure self-stigma related to various minorities and diseases by adding to each question what self-stigma is to be measured. Hence, the expression “COVID-19 infected persons” was added to each item in this study. SSS-S comprises nine items rated on a 4-point Likert scale from 1 (strongly disagree) to 4 (strongly agree). A higher total score indicates a more severe self-stigma.

#### Covariates

Demographic data included age groups (20–29, 30–39, 40–49, 50–59, 60–69, 70–79, 80–89, ≥ 90 years), sex (male, female, other), educational level (high school education or lower, higher than high school), income level (based on the definitions by the Ministry of Health, Labor and Welfare [38]); (low, < 3 million JPY; medium, < 10 million JPY; high, ≥ 10 million JPY; unknown or refused to answer), residence (alone, co-residing), presence of physical comorbidities, and the presence of psychiatric history.

Clinical information regarding COVID-19 infection: duration after infection (less than one month, less than three months, less than six months, six months and over), whether hospitalized, and the presence of physical sequelae symptoms after COVID-19 infection, were also collected. All variables except the age group and sex were processed to binary numbers. Sex was converted to male or others for avoiding multicollinearity.

### Statistical analysis

The characteristics of the participants in this study were primarily confirmed by calculating the mean and standard deviation (SD) for continuous variables, and the number and proportion for categorical variables. Modified Poisson regression analysis was applied to explore the association between exposure and respective outcome variables after adjusting the covariates. Following covariates were included in the model, based on the view whether they influenced both exposure and outcome variables: duration after infection [20, 39], hospitalization [14, 20, 40], presence of physical sequelae symptoms after COVID-19 infection [21, 41, 42], income level [43–45], residence [13, 46], presence of physical comorbidities [17, 18, 42], and presence of psychiatric history [18, 20, 47]. Demographic information such as age group, sex, and educational level was also included in the model.

*P*-values less than 0.05 were considered statistically significant. Analyses were performed using Stata version 17 (College Station, TX: Stata Corp LLC).

## Results

### Participant characteristics

**Fig. 1:**
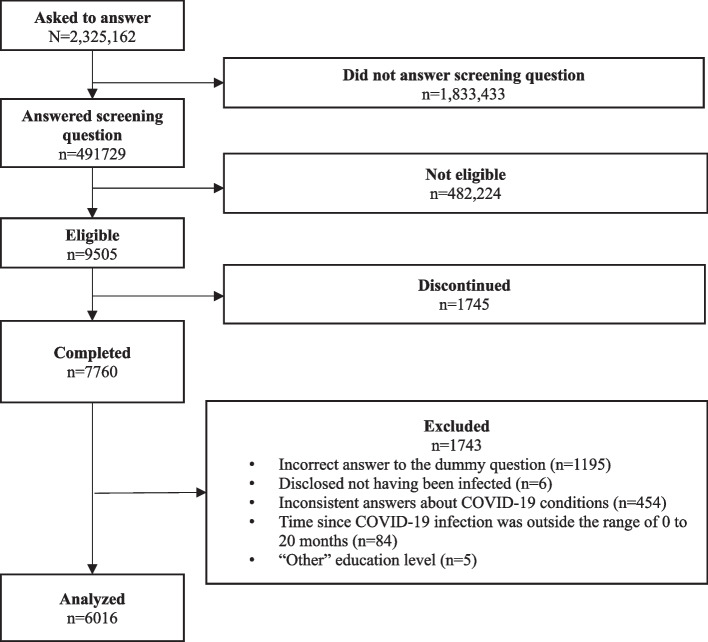
Flow chart of the selection process of participants

Figure 1 shows the process of participant selection. Out of 9505 eligible individuals, 7760 completed the questionnaire. After excluding 1743 participants for incorrect or contradictory answers to the dummy question, 6016 were finally analyzed.

The demographic data for 6016 participants with COVID-19 infection are shown in Table 1. The highest rate of affection was among 30–49 age group, while age 80–90 group had the least affected rate of infection. The proportion of females was higher than males or others. In terms of other demographic factors, 77.48% were higher than high school educated, 60.27% were classified as belonging to medium level of income, and 80.63% were living with someone. Individuals with psychiatric history were 17.73%, and those with physical comorbidity were 46.30%.

**Table 1:** Participant characteristics

	Total (*n* = 6016)	
**Age group**		
20–29	*n* = 1192	19.81%
30–39	*n* = 1505	25.02%
40–49	*n* = 1581	26.28%
50–59	*n* = 1189	19.76%
60–69	*n* = 436	7.25%
70–79	*n* = 101	1.68%
80–89	*n* = 7	0.12%
≥ 90	*n* = 5	0.08%
**Sex**		
Female	*n* = 3441	57.20%
Male	*n* = 2555	42.47%
Others	*n* = 20	0.33%
**Educational level**		
Higher than high school educated	*n* = 4661	77.48%
**Income level**		
Low	*n* = 842	14.00%
Medium	*n* = 3626	60.27%
High	*n* = 988	16.42%
Unknown/r refused to answer	*n* = 560	9.31%
**Living together**		
Yes	*n* = 4851	80.63%
**Psychiatric history**		
Yes	*n* = 1067	17.73%
**Physical comorbidity**		
Yes	*n* = 2788	46.30%
**Hospitalized**		
Yes	*N* = 1740	24.43%
**Persistent symptoms**		
Yes	*n* = 2345	38.98%
**Duration after infection**	mean = 4.5	± 4.37
Less than 1 month	*n* = 1138	18.92%
Less than 3 months	*n* = 1443	23.99%
Less than 6 months	*n* = 1369	22.76%
6 months or more	*n* = 2066	34.34%
**Depression (PHQ-9)**	mean = 5.19	± 5.91
≥ 10	*n* = 1196	19.88%
**Anxiety (GAD-7)**	mean = 3.34	± 4.71
≥ 10	*n* = 690	11.47%
**Self-stigma (SSS-S)**	mean = 9.21	± 7.40
Threat of life due to COVID-19 infection	*n* = 1184	19.68%
Helplessness due to COVID-19 infection	*n* = 1681	27.94%
Blaming the third party who did not restrain from going outside	*n* = 905	15.04%
Blaming themselves for their COVID-19 infection	*n* = 783	13.02%
Worry about spreading to others	*n* = 3113	51.75%

*PHQ-9* Patient Health Questionnaire-9, *GAD-7* The Generalized Anxiety Disorder-7, *SSS-S* Self-Stigma Scale-Short

Regarding COVID-19 condition, 24.43% were hospitalized for COVID-19 infection, and 38.98% have had some persistent symptoms. More than 1/3 of the participants (34.34%) had post COVID -19 infection 6 months or more.

The mean PHQ-9 score was 5.19 (± 5.91), and the proportion of participants with a score over 10 was 19.88%. The mean GAD-7 score was 3.34 (± 4.71), and 11.47% participants obtained a score over 10.

More than half of the patients (51.75%) were worried about spreading infection to others, while 27.94% felt helpless due to COVID -19 infection.

### Attitudes toward COVID-19 infection related to depression and anxiety

For depression, the threat of life due to COVID-19 infection (adjusted IRR = 1.23, 95% CI = 1.11–1.38, *p* < 0.001), helplessness regarding COVID-19 infection (adjusted IRR = 1.21, 95% CI = 1.09–1.33, *p* < 0.001), blaming themselves for their COVID-19 infection (adjusted IRR = 1.24, 95% CI = 1.1–1.39, *p* < 0.001), self-stigma (adjusted IRR = 1.05, 95% CI = 1.04–1.05, *p* < 0.001) were significantly associated with depression, independent of covariates (Table 2). VIF was 1.04—2.28.

**Table 2:** Attitude towards COVID-19 infection related to depression

	**Depression (PHQ-9)**			**Anxiety (GAD-7)**		
	**Adjusted** **IRR**	**(95% CI)**	***p***	**Adjusted** **IRR**	**(95% CI)**	***p***
Threat of life due to COVID-19 infection						
Not strongly experienced	1.00			1.00		
Strongly experienced	**1.23**	**(1.11—1.38)**	**< 0.001**	**1.35**	**(1.15—1.58)**	**< 0.001**
Helplessness due to COVID-19 infection						
Not strongly experienced	1.00			1.00		
Strongly experienced	**1.21**	**(1.09—1.33)**	**< 0.001**	**1.23**	**(1.07—1.42)**	**0.003**
Blaming the third party who did not restrain from going outside						
Not strongly experienced	1.00			1.00		
Strongly experienced	1.08	(0.96—1.21)	0.19	**1.21**	**(1.04—1.41)**	**0.01**
Blaming themselves for their COVID-19 infection						
Not strongly experienced	1.00			1.00		
Strongly experienced	**1.24**	**(1.1—1.39)**	**< 0.001**	**1.21**	**(1.03—1.43)**	**0.02**
Worry about spreading to others						
Not strongly experienced	1.00			1.00		
Strongly experienced	0.93	(0.84—1.03)	0.15	0.87	(0.75—1.01)	0.06
Self-stigma (SSS-S)	**1.05**	**(1.04—1.05)**	**< 0.001**	**1.06**	**(1.05—1.07)**	**< 0.001**
Hospitalized						
No	1.00			1.00		
Yes	1.05	(0.95—1.16)	0.37	1.05	(0.91—1.21)	0.49
Persistent symptoms						
No	1.00			1.00		
Yes	**2.29**	**(2.05—2.57)**	**< 0.001**	**2.19**	**(1.87—2.56)**	**< 0.001**
Duration after infection						
Less than 1 month	1.00			1.00		
Less than 3 months	1.05	(0.91—1.21)	0.49	**1.27**	**(1.03—1.57)**	**0.03**
Less than 6 months	1.22	**(1.06—1.4)**	**0.004**	**1.51**	**(1.23—1.85)**	**< 0.001**
6 months or more	1.11	(0.97—1.27)	0.14	**1.37**	**(1.13—1.67)**	**0.001**
Psychiatric history						
No	1.00			1.00		
Yes	**1.98**	**(1.79—2.19)**	**< 0.001**	**2.46**	**(2.13—2.84)**	**< 0.001**
Physical comorbidity						
No	1.00			1.00		
yes	**1.23**	**(1.11—1.35)**	**< 0.001**	1.13	(0.99—1.3)	0.07
Sex						
Others	1.00			1.00		
Male	0.98	(0.88**—**1.08)	0.71	0.93	(0.81—1.07)	0.33
Age group	**0.82**	**(0.79—0.86)**	**< 0.001**	**0.83**	**(0.78—0.88)**	**< 0.001**
Educational level						
High school educated or lower	1.00			1.00		
Higher than high school educated	0.94	(0.85—1.05)	0.29	1.02	(0.87—1.19)	0.85
Living together						
No	1.00			1.00		
Yes	**0.85**	**(0.76—0.96)**	**0.008**	0.89	(0.75—1.05)	0.18
Income level						
Low	1.00			1.00		
Medium	0.83	**(0.74—0.94)**	**0.004**	0.84	(0.7—1)	0.052
High	0.76	**(0.63—0.9)**	**0.002**	0.78	(0.61—1)	0.052
Unknown/ refused to answer	0.78	**(0.64—0.95)**	**0.01**	0.93	(0.71—1.2)	0.56

*IRR* Incidence Rate Ratio, *CI* Confidence Interval, *PHQ-9* Patient Health Questionnaire-9, *GAD-7* The Generalized Anxiety Disorder-7, *SSS-S* Self-Stigma Scale-Short

For anxiety, threat of life due to infection (adjusted IRR = 1.35, 95% CI = 1.15–1.58, *p* < 0.001), helplessness regarding COVID-19 infection (adjusted IRR = 1.23, 95% CI = 1.07–1.42, *p* = 0.003), blaming the third party who did not restrain from going outside (adjusted IRR = 1.21, 95% CI = 1.04–1.41, *p* = 0.01), blaming themselves for their COVID-19 infection (adjusted IRR = 1.21, 95% CI = 1.03–1.43, *p* = 0.02), self-stigma (adjusted IRR = 1.06, 95% CI = 1.05–1.07, *p* < 0.001) were associated with anxiety, independent of covariates (Table 2). VIF was 1.05—2.28.

Worry about spreading the infection to others was not significantly associated with either PHQ-9 or GAD-7 scores.

## Discussion

This cross-sectional study investigated the association between attitudes toward COVID-19 infection and psychiatric symptoms such as depression and anxiety, which are common sequelae of COVID-19 among COVID-19 recovered individuals. Our findings revealed that strong attitude towards COVID-19 infection, such as the threat of life and helplessness regarding COVID-19 infection, blaming self for their COVID-19 infection, and self-stigma were associated with depression and anxiety in COVID-19 recovered patients. Blaming the third party who did not restrain from going outside was associated with anxiety.

Strong threat of life due to infection was associated with depression and anxiety. Although this has already been identified by a qualitative study [27], our study is the first to quantitatively show the relationships. In general, depression and anxiety are likely to occur after life-threatening traumatic events [48, 49]. Since hospitalization status was adjusted in this study, the subjective threat of life is interpreted as an independent factor, separate from the actual danger to life. COVID-19 recovered patients tend to perceive the infection as a threat to life even when most of them are not considered in immediate danger by professionals. They recognize the prognosis of COVID-19 infection as unclear [23], and are prone to be driven into a corner during isolation [13, 50, 51]; any sudden changes in their conditions are difficult to notice because of isolation.

A strong tendency to self-blame for one’s COVID-19 infection was also associated with depression and anxiety, as confirmed by previous qualitative studies [24, 25]. This might also be a reason for depression and anxiety post recovery. In general, self-blaming is a distinguishing factor predicting depression [52]. It is presumed that when people blame themselves, pessimistic predictions about how they will be evaluated by their surroundings, such as being blamed by others, arise. Such catastrophic future predictions are known to affect anxiety in general populations [53]. These relationships between self-blaming and depression and anxiety might also apply to COVID-19 recovered patients.

A strong feeling of helplessness associated with COVID-19 infection contributes to depression and anxiety. This helplessness has not been reported in previous studies, while approximately 27% were found to suffer this cognition that affected depression and anxiety among COVID-19 recovered patients. The studies of the general population during COVID-19 pandemic provided contrary results to those of our study: strong feeling of helplessness regarding COVID-19 infection was associated with low anxiety as per the theory of motivated helplessness [54, 55].The idea of reliving painful experiences induced by COVID-19 infection, might be challenging to accept and result in anticipatory anxiety of reinfection as inevitable. Helplessness is a common cognitive distortion based on lacking the locus of control and leading to depression and anxiety [56, 57]. Since people were still being infected with COVID-19 despite various precautions, such as lockdown, masks, washing, and vaccination, they might perceive reinfection as unavoidable.

COVID-19-related self-stigma also contributed to depression and anxiety in COVID-19 recovered patients. Our results supported previous studies indicating relationships between COVID-19-related self-stigma and psychiatric symptoms such as depression, anxiety, insomnia, and overall mental health [28, 29, 58]. Self-stigma is suggested to activate low self-esteem resulting in depression, as with patients with other several illnesses [59–61]. Furthermore, COVID-19 recovered patients with high self-stigma are probably concerned about negative impressions from others because their self-stigma is activated by internalizing public stigma [62]. These concerns were risk factors for anxiety in general [63].

Blaming the third party, policies or those who did not exercise restraint, was associated with anxiety, but not depression. The general population during COVID-19 pandemic tended to blame the government [64], and such cognition of blaming contributes to anxiety about COVID-19 safety [65, 66]. This may also be true for patients recovered from COVID-19. Critical thinking about policies and morals is shaped by recognizing that they violate a safe social environment [67]. Various risks associated with COVID-19 pandemic induce anxiety in the general population [68]. COVID-19 recovered patients often fear reinfection because a high incidence of reinfection [69–72]. Contrarily, the tendency to blame others did not relate to depression in general [52].

The worry about spreading the infection to others was not related to depression or anxiety. More than 50% of the participants had this attitude to COVID-19 infection is highly infectious and dangerous [1]. Therefore, it may be an extremely common and healthy reaction to have such a worry. The reason why this attitude did not find to be associated with depression and anxiety is unclear. The attitude reflects not at the time of response but at the time of infection in this study. Such attitude might be temporal and have no affection to chronic psychiatric symptoms.

COVID-19 recovered patients with low-income levels had high depression and anxiety. This finding was consistent with previous studies [43, 44, 73]. Depression and anxiety in the older age group were lower. The same trend was observed in several studies of COVID-19 recovered patients [12, 15, 16, 74]. Physical comorbidity was associated with depression and tend to be associated with anxiety although not significant, as a systematic review indicates that any comorbidity before COVID-19 infection was related to depression and anxiety [16]. Also, some studies suggest that the baseline Charlson comorbidities index predicts depression and anxiety after infection [17, 18]. In addition, the relationship between a psychiatric history and depression and anxiety was also reported by previous studies [18, 20, 75]. As for the duration after infection, depression remained over time except at the 6-month time point, and anxiety was higher at over one month than at less than one month. The results are in line with previous studies indicating that depression and anxiety increase from 3–12 months [4, 14]. Physical symptoms as sequelae were associated with depression and anxiety, as observed in previous studies [21, 41]. Those who living alone have depression and anxiety more frequently than those who living together. This is consistent with the survey that loneliness and social withdrawal affect depression in COVID-19 patients [13]. Living alone may not be associated with anxiety because of offsetting of negative and positive impacts: living with others induces worry about spreading infection to others as well as mental stability through cohabitation [46]. Hospitalization was not related to depression or anxiety in this study and reports on this association are inconsistent across studies [2, 14, 20]. This discrepancy may be seen because standards and facilities for hospitalization vary from country to country and the time of infection.

This study has some limitations which must be considered. First, concerns the sampling bias. Participants were recruited via the online research company; hence, they were limited to those who were able to access online systems. Over 90% of participants were under the age of 60, despite the higher percentage of COVID-19 infected patients being those above 60 years of age in Japan [76]. Also, COVID-19 recovered patients whose sequelae were too severe to complete the questionnaire, could not participate in this study. Therefore, our results may not apply to elderly and individuals with high severity. Second, all variables were self-reported. Thus, variables such as diagnosis, the presence of infection, and treatment conditions may have been limited in accuracy. However, the proportion of COVID-19 recovered patients who agreed to participate in this study is similar to the proportion of COVID-19 infected patients in Japan [76]. In addition, since the attitudes toward COVID-19 were generated based on the experience of one COVID-19 recovered patient, these were not investigated comprehensively; nor were they validated well. Third, the causal relationship between the study variables remains unclear because of the cross-sectional design. Whether changes in negative attitudes improve depression and anxiety is uncertain. Forth, there are potential confounding variables not adjusted in this study. Despite these limitations, this study has the advantage of studying a large sample and revealing the status of Japanese COVID-19 recovered patients, including those not linked to medical care.

## Conclusion

This study revealed that negative attitudes, such as threat to life and helpless due to COVID-19 infection, blaming a third party or oneself for one’s infection were related with depression and anxiety, independent of biological factors or baseline comorbidity. Further studies should be performed to confirm the causal relationships between such attitudes and depression and anxiety with the longitudinal or clinical trial design.

## Data Availability

The data used in this study will be made available to the corresponding author upon reasonable request.

## Abbreviations

PHQ-9: The Patient Health Questionnaire-9
GAD-7: The Generalized Anxiety Disorder-7
SSS-S: Self-Stigma Scale-Short

## References

[1] World Health Organization. A clinical case definition of post COVID-19 condition by a Delphi consensus, 6 October 2021. 2021. p. 27. Available from: https://apps.who.int/iris/handle/10665/345824.

[2] Groff D, Sun A, Ssentongo AE, Ba DM, Parsons N, Poudel GR (2021). Short-term and Long-term Rates of Postacute Sequelae of SARS-CoV-2 Infection. JAMA Netw Open.

[3] Morin L, Savale L, Pham T, Colle R, Figueiredo S, Harrois A (2021). Four-Month Clinical Status of a Cohort of Patients after Hospitalization for COVID-19. JAMA.

[4] Huang L, Yao Q, Gu X, Wang Q, Ren L, Wang Y (2021). Articles 1-year outcomes in hospital survivors with COVID-19: a longitudinal cohort study. Lancet..

[5] Taquet M, Geddes JR, Husain M, Luciano S, Harrison PJ. Articles 6-month neurological and psychiatric outcomes in 236 379 survivors of COVID-19 : a retrospective cohort study using electronic health records. Lancet Psychiatry. Available from: 10.1016/S2215-0366(21)00084-5 10.1016/S2215-0366(21)00084-5 PMC8023694 33836148

[6] Xie Y, Xu E, Al-Aly Z (2022). Risks of mental health outcomes in people with covid-19: cohort study. BMJ.

[7] Taquet M, Luciano S, Geddes JR, Harrison PJ (2021). Bidirectional associations between COVID-19 and psychiatric disorder: retrospective cohort studies of 62 354 COVID-19 cases in the USA. Lancet Psychiatry..

[8] Xie Y, Xu E, Al-Aly Z (2022). Risks of mental health outcomes in people with covid-19: cohort study. BMJ..

[9] Knapp M, Wong G (2020). Economics and mental health: the current scenario. World Psychiatry.

[10] König H, König H-H, Konnopka A (2020). The Excess Costs of Depression: A Systematic Review and Meta-Analysis. Epidemiol Psychiatr Sci.

[11] Konnopka A, König H (2020). Economic Burden of Anxiety Disorders: A Systematic Review and Meta-Analysis. Pharmacoeconomics.

[12] Gramaglia C, Gambaro E, Bellan M, Balbo PE, Baricich A, Sainaghi PP (2021). Mid-term Psychiatric Outcomes of Patients Recovered From COVID-19 From an Italian Cohort of Hospitalized Patients. Front Psychiatry..

[13] Crook H, Raza S, Nowell J, Young M, Edison P (2021). Long covid - Mechanisms, risk factors, and management. BMJ.

[14] Premraj L, Kannapadi NV, Briggs J, Seal SM, Battaglini D, Fanning J (2022). Mid and long-term neurological and neuropsychiatric manifestations of post-COVID-19 syndrome: A meta-analysis. J Neurol Sci..

[15] Mazza MG, De Lorenzo R, Conte C, Poletti S, Vai B, Bollettini I (2020). Anxiety and depression in COVID-19 survivors: Role of inflammatory and clinical predictors. Brain Behav Immun..

[16] Thye AY-K, Law JW-F, Tan LT-H, Pusparajah P, Ser H-L, Thurairajasingam S, Letchumanan V (2022). Psychological Symptoms in COVID-19 Patients : Insights into Pathophysiology and Risk Factors of Long COVID-19. Biology (Basel)..

[17] Wong AW, Shah AS, Johnston JC, Carlsten C, Ryerson CJ (2020). Patient-reported outcome measures after COVID-19: A prospective cohort study. Eur Respir J..

[18] Tenforde MW, Kim SS, Lindsell CJ, Billig Rose E, Shapiro NI, Files DC (2020). Symptom Duration and Risk Factors for Delayed Return to Usual Health Among Outpatients with COVID-19 in a Multistate Health Care Systems Network — United States, March-June 2020. Morb Mortal Wkly Rep.

[19] Mannan A, Mehedi HMH, Chy NUHA, Qayum MO, Akter F, Rob MA (2021). A multi-centre, cross-sectional study on coronavirus disease 2019 in Bangladesh: clinical epidemiology and short-term outcomes in recovered individuals. New Microbes New Infect..

[20] Schou TM, Joca S, Wegener G, Bay-Richter C (2021). Psychiatric and neuropsychiatric sequelae of COVID-19 – A systematic review. Brain Behav Immun..

[21] de Graaf MA, Antoni ML, ter Kuile MM, Arbous MS, Duinisveld AJF, Feltkamp MCW (2021). Short-term outpatient follow-up of COVID-19 patients: A multidisciplinary approach. EClinicalMedicine..

[22] Hüfner K, Tymoszuk P, Ausserhofer D, Sahanic S, Pizzini A, Rass V (2022). Who Is at Risk of Poor Mental Health Following Coronavirus Disease-19 Outpatient Management?. Front Med.

[23] Beck K, Vincent A, Becker C, Keller A, Cam H, Schaefert R (2021). Prevalence and factors associated with psychological burden in COVID-19 patients and their relatives: A prospective observational cohort study. PLoS One..

[24] Heiberg KE, Heggestad AKT, Jøranson N, Lausund H, Breievne G, Myrstad M (2022). ‘Brain fog’, guilt, and gratitude: experiences of symptoms and life changes in older survivors 6 months after hospitalisation for COVID-19. Eur Geriatr Med..

[25] Alkaissi A, Zaben F, Abu-Rajab M, Alkony M (2022). m. BMC Public Health.

[26] Sun N, Wei L, Wang H, Wang X, Gao M, Hu X (2021). Qualitative study of the psychological experience of COVID-19 patients during hospitalization. J Affect Disord.

[27] Moradi Y, Mollazadeh F, Karimi P, Hosseingholipour K, Baghaei R (2020). Psychological disturbances of survivors throughout COVID-19 crisis: a qualitative study. BMC Psychiatry.

[28] Kang EK, Lee SY, Kim MS, Jung H, Kim KH, Kim KN (2021). The Psychological Burden of COVID-19 Stigma: Evaluation of the Mental Health of Isolated Mild Condition COVID-19 Patients. J Korean Med Sci.

[29] Mahmoudi H, Saffari M, Movahedi M, Sanaeinasab H, Rashidi-Jahan H, Pourgholami M (2021). A mediating role for mental health in associations between COVID-19-related self-stigma, PTSD, quality of life, and insomnia among patients recovered from COVID-19. Brain Behav.

[30] Campo-Arias A, Pedrozo-Pupo JC, Caballero-Domínguez CC (2022). Relation of perceived discrimination with depression, insomnia and post-traumatic stress in COVID-19 survivors. Psychiatry Res..

[31] von Elm E, Altman DG, Egger M, Pocock SJ, Gøtzsche PC, Vandenbroucke JP (2008). The Strengthening the Reporting of Observational Studies in Epidemiology (STROBE) statement: guidelines for reporting observational studies. J Clin Epidemiol.

[32] Kroenke K, Spitzer RL, Williams JBW (2001). The PHQ-9: Validity of a brief depression severity measure. J Gen Intern Med.

[33] Muramatsu K, Miyaoka H, Kamijima K, Muramatsu Y, Tanaka Y, Hosaka M (2018). Performance of the Japanese version of the Patient Health Questionnaire-9 (J-PHQ-9) for depression in primary care. Gen Hosp Psychiatry..

[34] Spitzer RL, Kroenke K, Williams JBW, Lowe B (2006). A brief measure for assessing generalized anxiety disorder. Arch Intern Med.

[35] Muramatsu K, Miyaoka H, Ueshima K, Muramatsu Y, Fuse K, Yoshimine H (2009). Validation and utility of a Japanese version of the GAD-7. Panminerva Medica 20th World Congr Psychosom Med Abstr B..

[36] Wu TH, Chang CC, Chen CY, Der WJ, Lin CY (2015). Further psychometric evaluation of the Self-Stigma Scale-Short: Measurement invariance across mental illness and gender. PLoS ONE.

[37] Kato A, Takada M, Hashimoto H (2014). Reliability and validity of the Japanese version of the Self-Stigma Scale in patients with type 2 diabetes.

[38] The ministry of health labor and Welfare. Income of each household. 2018. Available from: https://www.mhlw.go.jp/toukei/saikin/hw/k-tyosa/k-tyosa19/dl/03.pdf

[39] Lambert AE, Hu Y, Magee JC, Beadel JR, Teachman BA (2014). Thought suppression across time: Change in frequency and duration of thought recurrence. J Obs Compuls Relat Disord.

[40] Zarębska-Michaluk D, Rzymski P, Moniuszko-Malinowska A, Brzdęk M, Martonik D, Rorat M (2022). Does Hospitalization Change the Perception of COVID-19 Vaccines among Unvaccinated Patients?. Vaccines.

[41] Tomasoni D, Bai F, Castoldi R, Barbanotti D, Falcinella C, Mulè G (2021). Anxiety and depression symptoms after virological clearance of COVID-19: A cross-sectional study in Milan. Italy J Med Virol.

[42] Hagger MS, Koch S, Chatzisarantis NLD, Arat S, Araújo-soares V, Benyamini Y (2017). The Common Sense Model of Self-Regulation : Meta-Analysis and Test of a Process Model. Psychol Bull.

[43] Perlis RH, Santillana M, Ognyanova K, Green J, Druckman J, Lazer D (2021). Factors Associated with Self-reported Symptoms of Depression among Adults with and without a Previous COVID-19 Diagnosis. JAMA Netw Open.

[44] Sareen J, Afifi TO, McMillan KA, Asmundson GJG (2011). Relationship Between Household Income and Mental Disorders. Arch Gen Psychiatry.

[45] Irigoyen-Camacho ME, Velazquez-Alva MC, Zepeda-Zepeda MA, Cabrer-Rosales MF, Lazarevich I, Castaño-Seiquer A (2020). Effect of income level and perception of susceptibility and severity of covid-19 on stay-at-home preventive behavior in a group of older adults in Mexico City. Int J Environ Res Public Health.

[46] Sahashi Y, Endo H, Sugimoto T, Nabeta T, Nishizaki K, Kikuchi A (2021). Worries and concerns among healthcare workers during the coronavirus 2019 pandemic: A web-based cross-sectional survey. Humanit Soc Sci Commun..

[47] Özdel K, Taymur I, Guriz SO, Tulaci RG, Kuru E, Turkcapar MH (2014). Measuring cognitive errors using the Cognitive Distortions Scale (CDS): Psychometric properties in clinical and non-clinical samples. PLoS One..

[48] Vrana S, Lauterbach D (1994). Prevalence of traumatic events and post-traumatic psychological symptoms in a nonclinical sample of college students. J Trauma Stress.

[49] Gabbe BJ, Simpson PM, Cameron PA, Ponsford J, Lyons RA, Collie A (2017). Long-term health status and trajectories of seriously injured patients: A population-based longitudinal study. PLoS Med.

[50] Fernández RS, Crivelli L, Guimet NM, Allegri RF, Pedreira ME (2020). Psychological distress associated with COVID-19 quarantine: Latent profile analysis, outcome prediction and mediation analysis. J Affect Disord.

[51] Kowalski E, Schneider A, Zipfel S, Stengel A, Graf J (2021). SARS-CoV-2 Positive and Isolated at Home: Stress and Coping Depending on Psychological Burden. Front Psychiatry.

[52] Lythe KE, Moll J, Gethin JA, Workman CI, Green S, Ralph MAL (2015). Self-blame-selective hyperconnectivity between anterior temporal and subgenual cortices and prediction of recurrent depressive episodes. JAMA Psychiat.

[53] Rapee RM, Heimberg RG (1997). A Cognitive-Behavioral Model of Anxiety in Social Phobia. Behav Res Ther.

[54] Lifshin U, Mikulincer M, Kretchner M (2022). Motivated Helplessness in the Coronavirus Pandemic: Experimental Evidence that Perceived Helplessness to Avoid the Virus Reduces Fear of Covid-19. J Soc Clin Psychol.

[55] Lifshin U, Mikulincer M (2021). Further evidence for motivated helplessness in the context of the COVID-19 outbreak: the case of Argentina before and during the pandemic. J Soc Psychol.

[56] Miller WR, Seligman MEP, Kurlander HM (1975). Learned helplessness, depression, and anxiety. J Nerv Ment Dis.

[57] Fincham FD, Hokoda A, Sanders R (1989). Learned Helplessness, Test Anxiety, and Academic Achievement: A Longitudinal Analysis. Child Dev.

[58] Alkathiri MA, Almohammed OA, Alqahtani F, Alruthia Y (2022). Associations of Depression and Anxiety with Stigma in a Sample of Patients in Saudi Arabia Who Recovered from COVID-19. Psychol Res Behav Manag.

[59] Allabadi H, Alkaiyat A, Alkhayyat A, Hammoudi A, Odeh H, Shtayeh J (2019). Depression and anxiety symptoms in cardiac patients: A cross-sectional hospital-based study in a Palestinian population 11 Medical and Health Sciences 1117 Public Health and Health Services 11 Medical and Health Sciences 1103 Clinical Sciences. BMC Public Health.

[60] Kato A, Fujimaki Y, Fujimori S, Isogawa A, Onishi Y, Suzuki R (2020). How self-stigma affects patient activation in persons with type 2 diabetes: A cross-sectional study. BMJ Open.

[61] Phelan SM, Griffin JM, Jackson GL, Zafar SY, Stahre M, Nelson D (2018). Stigma, perceived blame, self-blame, and depressive symptoms in men with colorectal cancer. Psychooncology.

[62] Vogel DL, Bitman RL, Hammer JH, Wade NG (2013). Is stigma internalized? The longitudinal impact of public stigma on self-stigma. J Couns Psychol.

[63] Clark DM, Wells A, Heimberg RG, Liebowitz MR, Hope DA, Schneier FR (1995). A cognitive model of social phobia. Social phobia: Diagnosis, assessment, and treatment.

[64] Inoue Y, Okita T (2021). Coronavirus Disease and the Shared Emotion of Blaming Others: Reviewing Media Opinion Polls During the Pandemic. J Epidemiol.

[65] Hardy LJ, Mana A, Mundell L, Neuman M, Benheim S, Otenyo E (2021). Who is to blame for COVID-19? Examining politicized fear and health behavior through a mixed methods study in the United States. PLoS One..

[66] Abadi D, Arnaldo I, Fischer A (2021). Anxious and Angry: Emotional Responses to the COVID-19 Threat. Front Psychol.

[67] Malle BF, Guglielmo S, Monroe AE (2014). A Theory of Blame. Psychol Inq.

[68] Eshel Y, Kimhi S, Marciano H, Adini B (2021). Components of Unrealistic Optimism of College Students: The Case of the COVID-19 Pandemic. Front Psychol..

[69] Malhotra S, Mani K, Lodha R, Bakhshi S, Mathur VP, Gupta P (2022). SARS-CoV-2 Reinfection Rate and Estimated Effectiveness of the Inactivated Whole Virion Vaccine BBV152 Against Reinfection among Health Care Workers in New Delhi. India JAMA Netw Open.

[70] Sotoodeh Ghorbani S, Taherpour N, Bayat S, Ghajari H, Mohseni P, Hashemi Nazari SS (2022). Epidemiologic characteristics of cases with reinfection, recurrence, and hospital readmission due to COVID-19: A systematic review and meta-analysis. J Med Virol.

[71] Chivese T, Matizanadzo JT, Musa OAH, Furuya-kanamori L, Islam N, Al-shebly R (2022). The prevalence of adaptive immunity to COVID-19 and reinfection after recovery – a comprehensive systematic review and meta-analysis. Pathog Glob Health..

[72] Alkaissi A, Zaben F, Abu-Rajab M, Alkony M (2022). Lived experiences of Palestinian patients with COVID-19: a multi-center descriptive phenomenological study of recovery journey. BMC Public Health..

[73] Ridley M, Rao G, Schilbach F, Patel V (2020). Poverty, depression, and anxiety: Causal evidence and mechanisms. Science..

[74] Cai X, Hu X, Ekumi IO, Wang J, An Y, Li Z (2020). Psychological Distress and Its Correlates Among COVID-19 Survivors During Early Convalescence Across Age Groups. Am J Geriatr Psychiatry.

[75] Hazumi M, Usuda K, Okazaki E, Kataoka M, Nishi D. Differences in the Course of Depression and Anxiety after COVID-19 Infection between Recovered Patients with and without a Psychiatric History: A Cross-Sectional Study. Int J Environ Res Public Health 2022;19(18):11316. Available from: 10.3390/ijerph191811316. 10.3390/ijerph191811316 PMC9517442 36141588

[76] Ministry of Health Labour and Welfare. Visualizing the data: information on COVID-19 infections. Available from: https://covid19.mhlw.go.jp/en. cited 2022 Sep 9

